# Platelet-rich plasma injection for acute Achilles tendon rupture: two-year follow-up of the PATH-2 randomized, placebo-controlled, superiority trial

**DOI:** 10.1302/0301-620X.104B11.BJJ-2022-0653.R1

**Published:** 2022-11-01

**Authors:** David J. Keene, Joseph Alsousou, Paul Harrison, Heather M. O’Connor, Susan Wagland, Susan J. Dutton, Philippa Hulley, Sarah E. Lamb, Keith Willett, on behalf of the PATH-2 Trial group

**Affiliations:** 1 Kadoorie Research Centre, Nuffield Department of Orthopaedics, Rheumatology and Musculoskeletal Sciences, University of Oxford, Oxford, UK; 2 Manchester Royal Infirmary, Manchester University NHS Foundation Trust, Manchester, UK; 3 Institute of Inflammation and Ageing, University of Birmingham, Birmingham, UK; 4 Oxford Clinical Trials Research Unit, Centre for Statistics in Medicine, Nuffield Department of Orthopaedics, Rheumatology and Musculoskeletal Sciences, University of Oxford, Oxford, UK; 5 Botnar Research Centre, Nuffield Department of Orthopaedics, Rheumatology and Musculoskeletal Sciences, University of Oxford, Oxford, UK; 6 College of Medicine and Health, University of Exeter, Exeter, UK

**Keywords:** Platelet-rich plasma, Achilles tendon, Tendon rupture, Randomized controlled trial, acute Achilles tendon rupture, platelet-rich plasma (PRP) injection, platelet-rich plasma (PRP), Achilles Tendon Rupture Score (ATRS), platelets, placebo injection, ATRS scores, leg injury, re-ruptures, anticoagulation therapy

## Abstract

**Aims:**

To determine whether platelet-rich plasma (PRP) injection improves outcomes two years after acute Achilles tendon rupture.

**Methods:**

A randomized multicentre two-arm parallel-group, participant- and assessor-blinded superiority trial was undertaken. Recruitment commenced on 28 July 2015 and two-year follow-up was completed in 21 October 2019. Participants were 230 adults aged 18 years and over, with acute Achilles tendon rupture managed with non-surgical treatment from 19 UK hospitals. Exclusions were insertion or musculotendinous junction injuries, major leg injury or deformity, diabetes, platelet or haematological disorder, medication with systemic corticosteroids, anticoagulation therapy treatment, and other contraindicating conditions. Participants were randomized via a central online system 1:1 to PRP or placebo injection. The main outcome measure was Achilles Tendon Rupture Score (ATRS) at two years via postal questionnaire. Other outcomes were pain, recovery goal attainment, and quality of life. Analysis was by intention-to-treat.

**Results:**

A total of 230 participants were randomized, 114 to PRP and 116 to placebo. Two-year questionnaires were sent to 216 participants who completed a six-month questionnaire. Overall, 182/216 participants (84%) completed the two-year questionnaire. Participants were aged a mean of 46 years (SD 13.0) and 25% were female (57/230). The majority of participants received the allocated intervention (219/229, 96%). Mean ATRS scores at two years were 82.2 (SD 18.3) in the PRP group (n = 85) and 83.8 (SD 16.0) in the placebo group (n = 92). There was no evidence of a difference in the ATRS at two years (adjusted mean difference -0.752, 95% confidence interval -5.523 to 4.020; p = 0.757) or in other secondary outcomes, and there were no re-ruptures between 24 weeks and two years.

**Conclusion:**

PRP injection did not improve patient-reported function or quality of life two years after acute Achilles tendon rupture compared with placebo. The evidence from this study indicates that PRP offers no patient benefit in the longer term for patients with acute Achilles tendon rupture.

Cite this article: *Bone Joint J* 2022;104-B(11):1256–1265.

## Introduction

The most commonly ruptured tendon is the Achilles, and the incidence is rising as people maintain sporting participation into later life.^1,2^ Achilles tendon ruptures result in immediate and sustained limitations in weightbearing activities, leading to work incapacity and a prolonged period of inability to participate in sport.^3,4^

Platelet-rich plasma (PRP) is a popular intervention in sports medicine and orthopaedic practice for musculoskeletal soft-tissue injuries,^5^ and has received substantial public attention due to its promise as a regenerative therapy.^6^ PRP is an autologous blood product that provides a supraphysiological concentration of platelets, leucocytes, growth factors, and other bioactive proteins such as cytokines and chemokines for delivery to an injury site.^7^ PRP basic science studies have shown positive cellular and physiological effects on tendon healing under laboratory conditions, and although there are over 30 published clinical trials on PRP applications in musculoskeletal injuries, its efficacy remains uncertain.^8,9^

In the PATH-2 study, we aimed to determine the clinical efficacy of a standardized PRP preparation for acute non-surgically managed Achilles tendon ruptures in a randomized controlled trial.^10,11^ The rationale for PRP in Achilles ruptures is to improve the speed of healing, final quality of recovery, or ideally both. If a difference in speed of healing is evident, it would be observed when the tendon was in the initial recovery stages. The PATH-2 trial primary outcome measure at 24 weeks post-injury was timed to capture speed of recovery, and found no evidence of a difference in objective muscle function (heel-rise endurance test (HRET)) and patient-reported outcomes.^10^ However, Achilles tendon rupture prevents full return to function, sport, and work over a longer period, with an overall recovery time of greater than two years post-injury.^12,13^ Therefore, if PRP affects the quality of the recovering tendon, we could expect to see this in longer-term outcomes. In this study we conducted extended follow-up at two years post-randomization, to assess whether there is any beneficial effect of PRP on the quality of the recovering Achilles tendon in terms of level of long-term recovery of function.

## Methods

### Design

PATH-2 was a placebo-controlled, multicentre, superiority, randomized controlled two-arm parallel-group trial with blinded participants and outcome assessments. The trial was conducted at 19 hospitals in England and Wales, UK. The trial protocol and analysis plan have been published and are summarized below.^14,15^ The study was approved by the National Research Ethics Service Oxfordshire Committee A (reference 14/SC/1333) and overseen by an independent trial steering committee and data monitoring and ethics committee. Participants were recruited between 28 July 2015 and 18 September 2017, with two-year follow-up completed on 21 October 2019.

### Participants

Participants were adults aged 18 years and over with a clinical diagnosis, with or without diagnostic imaging, of complete acute mid-substance Achilles tendon rupture. To be eligible, patients had to be within 12 days of injury, able to walk unaided pre-injury, and non-surgically managed with a cast or boot. Patients were not eligible if they had a tendon rupture at the insertion or musculotendinous junction, previous major leg injury or deformity, diabetes mellitus, platelet or haematological disorder, current systemic corticosteroids, treatment doses of anticoagulation therapy, or other contraindicating conditions (lower limb gangrene/ulcers, peripheral vascular disease, hepatic or renal failure or dialysis, pregnant or breastfeeding, treatment with radiation or chemotherapy in previous three months, inadequate venous access). Development and amendments to the protocol have been described elsewhere.^10,14^

### Randomization

Participants were recruited in the trauma clinic and after providing written informed consent, baseline data were collected. Participants were then individually randomized 1:1 to an injection of PRP or placebo via the Oxford Clinical Trials Research Unit central 24-hour web-based randomization allocation system. Initial randomization was variable permuted blocks stratified by study site and age group (< 55/≥ 55 years). Monitoring identified that a technical issue resulted in the age group strata not being implemented. To ensure balance at the end of the study over stratification factors the system was switched to minimization using the existing randomized participants, including a probabilistic element (0.8). Minimization factors were study site and age group as originally intended.^16^ These amendments were approved by the trial oversight and ethics committees.

### Interventions

In the outpatient trauma clinic and within 12 days of injury, participants had blood withdrawn and received an injection in the gap in the ruptured tendon. Participants in the placebo group had 5 ml of venous blood withdrawn that was used for whole blood analysis. Participants assigned to PRP injection had 55 ml of venous blood withdrawn. A total of 5 ml was used for whole blood analysis, and 50 ml was used to produce 8 ml of leucocyte- and platelet-rich plasma using a specialized automated centrifuge (MAG 200 MAGELLAN Autologous Platelet Separator; Arteriocyte Medical Systems, USA) and a sterile disposable kit (MDK 300/300 to 1; Arteriocyte Medical Systems). All participants waited approximately 17 minutes after blood withdrawal before having the injection.

Participants lay face down on a treatment table and then the treating surgeon or specialist physiotherapist palpated the tendon to identify the tendon gap for injection. Ultrasound guidance was not mandated or recorded as this facility was not routinely available in the trauma clinic setting. After the injection site was cleaned, local anaesthetic (1 to 2 ml lidocaine) was injected into the skin, then the allocated injection (placebo or PRP) was performed in the centre of the tendon gap. For the PRP group, 4 ml of PRP was injected, and the remaining 4 ml was processed for laboratory analysis. The placebo injection used the same sized needle attached to an empty syringe inserted into the tendon gap, held in place for the duration of a PRP injection, and was withdrawn without injecting anything. All treating clinicians undertook study-specific training, used a step-by-step manual, and had access to a training video. Further details on PRP preparation and analysis are available elsewhere.^10,11,17^

Participants had the routine local non-surgical management for acute Achilles tendon rupture, apart from the standardized rehabilitation protocol. The rehabilitation requirements for the trial were that the ankle needed to be initially immobilized in an equinus position for at least three weeks post-injection, and avoidance of full-time ankle immobilization or non-weightbearing for longer than six weeks. All participants were referred to a physiotherapist for supervised rehabilitation.

Whole blood and PRP samples were analyzed in a central laboratory at the Institute of Inflammation and Ageing, University of Birmingham, Birmingham, UK. Cell counts were assessed using a Sysmex XN-1000 Haematology analyser (Sysmex UK, UK), providing three different platelet counts: impedance (PLT-I); optical (PLT-O); and fluorescent (PLT-F). Where possible, the PLT-F count was the preferred choice. Instrument performance was checked internally daily (XN Check; Sysmex UK) and externally monthly (UK NEQAS, UK).^18^ Platelet quality within fixed resting and activated samples (PAMfix; Platelet Solutions Ltd, UK) was analyzed by measuring the expression of the platelet-specific activation marker P-Selectin (CD62p) by flow cytometry (Accuri Flow cytometer; Becton Dickinson, UK). Growth factor concentrations (platelet-derived growth factor AB (PDGF-AB), insulin-like growth factor 1 (IGF-1), vascular endothelial growth factor (VEGF), basic fibroblast growth factor (FGFb), and transforming growth factor beta 1 (TGF-β1)) within the PRP were measured by enzyme-linked immunosorbent assay.

### Blinding

All participants were informed that up to 55 ml of venous blood would be taken. It was deemed unacceptable to take more venous blood than required for study participation, so the volume was different between the groups (55 ml for the PRP group and 5 ml for the placebo group). Injections were prepared away from participants and a dummy spin cycle was used for the placebo group if the centrifuge was nearby. Participants received the injection lying face down with instructions not to turn. Clinicians preparing or delivering the intervention could not be blinded.

### Outcome measures

The two-year follow-up study was conducted by postal questionnaires. The study team telephoned participants to improve response rates if there was no response by post. The patient-reported outcomes at earlier stages were by questionnaires completed face-to-face or by telephone at four, seven, 13, and 24 weeks post-randomization by a researcher at the recruiting centre.

Patient-reported symptoms and function were assessed using the Achilles Tendon Rupture Score (ATRS) (0 to 100, higher score better) (primary outcome for follow-up study).^19^ Functional limitations due to pain were assessed using the score from the pain-related ATRS (0 to 10, higher score better). Functional goal attainment was measured using the Patient-Specific Functional Scale (PSFS) (0 to 10, higher score better).^20,21^ Health-related quality of life was assessed using the 12-Item Short-Form Health Survey questionnaire (SF-12) v2 Health Survey (acute version, 0 to 100, higher score better).^22^ Participants were also asked about re-ruptures since the 24-week follow-up.

### Sample size

The primary outcome for the PATH-2 trial was the HRET, but the sample size also provided 90% power and 5% (two-sided) significance to detect an effect size of 0.5 in the principal secondary outcome, the ATRS, between the two treatment arms, based on a mean difference of 11 and a standard deviation (SD) of 21.4. A 58% response rate to the two-year follow-up (n = 124) would have provided 80% power at a 5% significance level to detect an effect size of 0.5 in the ATRS. With a more optimistic response rate of 79% (n = 168), the study would have had 90% power to detect a similar effect size (0.5) at the same significance level (5%). The actual response rate at two years was much higher at 84% (n = 182).

### Statistical analysis

The trial is reported following the Consolidated Standards of Reporting Trials (CONSORT) Statement and its related extensions.^23^ Analysis was undertaken on the modified intention-to-treat (ITT) population, that is: all randomized participants with available outcome data were analyzed in the groups to which they were allocated. All participants with baseline and at least one post-treatment questionnaire were included in the analysis. ATRS, PSFS, and SF-12 physical and mental components were analyzed using repeated measures linear mixed effects regression models incorporating all timepoints (as time-elapsed from randomization) as a random effect and adjusting for baseline values, stratification factors (study site and age group), and important prognostic factors (BMI, sex, and smoking status). Where the outcome measure was not normally distributed, non-parametric methods were used with no adjustment. As pain-related ATRS had hit the ceiling of ten points for many of the participants at 24 months, Spearman’s rank correlation coeficient was used to assess for differences between groups with no adjustment, and medians are presented. Compliance average causal effects (CACE) analyses were carried out for ATRS adjusting for treatment compliance and also for treatment compliance with high PRP. Consistency of effects across subgroups of age, BMI, smoking, and sex were assessed using Forest plots. Correlation between ATRS at two years and key blood parameters and bioactive factors for the participants who received the PRP intervention were explored using Pearson’s correlation. Statistical significance was set at a p-value < 0.05.

## Results

**Fig. 1:**
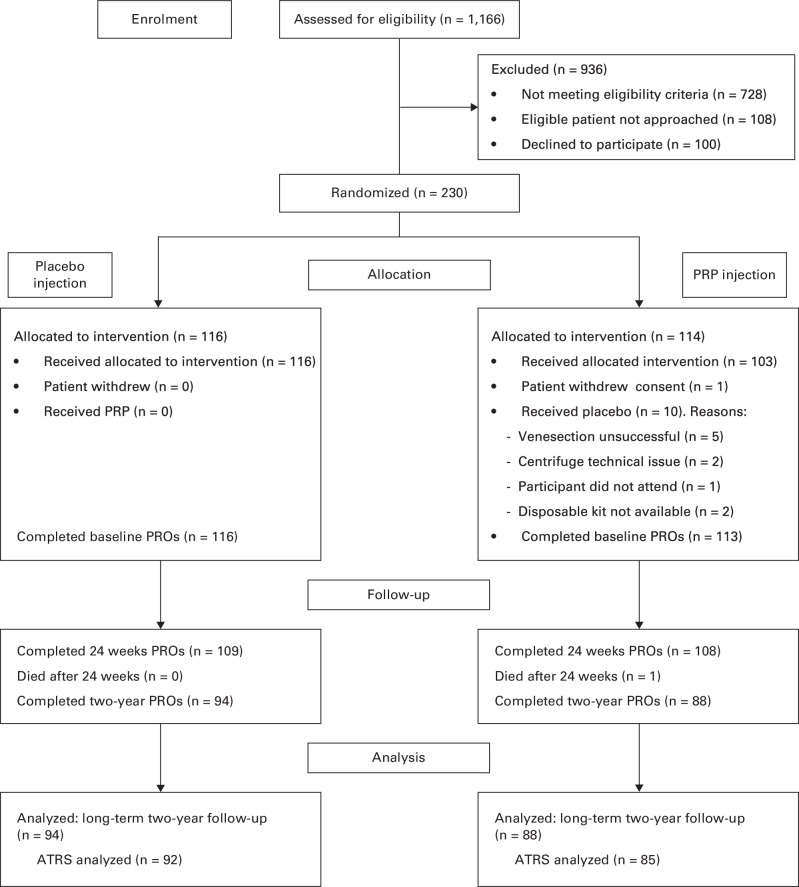
Flow of participants through the trial. ATRS, Achilles Tendon Rupture Score; PRO, patient-reported outcome; PRP, platelet-rich plasma.

Overall, 1,166 patients were screened for eligibility, and 230 consented to randomization (Figure 1).

Baseline characteristics were well balanced between the randomized groups and between responders and non-responders at two years’ follow-up (Table I). Participants had a mean age of 46 years (SD 13.0) and 57/230 (25%) were female. One participant withdrew consent prior to treatment.

**Table I.** Baseline demographic and clinical characteristics of randomized participants, summarized by treatment group and by whether they were a responder to the two-year follow-up.

Characteristic	PRP (n = 113)		Placebo (n = 116*)	
	Responder (n = 88)	Non-responder (n = 25)	Responder (n = 94)	Non-responder (n = 21)
Mean age, yrs (SD)	46.02 (13.91)	45.48 (13.40)	46.72 (12.10)	36.81 (9.00)
Sex (female), n (%)	19 (21.59)	6 (24)	28 (29.79)	3 (14.29)
Mean BMI, kg/m^2^ (SD)†	27.69 (5.16)	27.67 (5.85)	27.31 (4.21)	26.76 (4.38)
Mean time since injury, days (SD)	5.32 (2.98)	5.44 (2.90)	5.28 (3.15)	4.76 (2.84)
Mean alcohol consumption, units/week (SD)	9.73 (11.27)	10.52 (11.74)	10.68 (10.27)	10.00 (11.07)
Smoker, n (%)	10 (11.36)	4 (16.00)	9 (9.57)	4 (19.05)
Mean ATRS (SD)	13.70 (12.11)	13.72 (9.77)	11.36 (9.44)	12.57 (9.48)
Mean ATRS functional limitation due to pain score (SD)§	3.61 (3.45)	3.56 (3.06)	2.94 (3.00)	3.43 (3.16)
Mean PSFS (SD)¶	0.85 (1.04)	0.69 (0.84)	0.84 (1.21)	0.57 (0.65)
Mean SF-12 (SD)**				
Pre-injury Physical Component	53.33 (8.23)	54.03 (8.86)	52.45 (9.47)	53.70 (8.11)
Pre-injury Mental Component	55.51 (6.87)	46.07 (13.95)	54.44 (8.46)	50.83 (8.61)
Post-injury Physical Component	29.51 (6.91)	34.22 (6.97)	29.73 (7.57)	28.36 (6.43)
Post-injury Mental Component	49.22 (12.46)	40.68 (11.85)	49.85 (12.49)	46.59 (10.97)

* One participant died before the 24-month timepoint and therefore did not have the opportunity to respond.

† Data were not available for two participants in the placebo group.

‡ ATRS: scores were from 0 to 100, with 0 indicating “major limitations” and 100 indicating “no limitations”.

§ ATRS pain: Scores were from 0 to 10, with 0 indicating “major limitations” and 10 indicating “no limitations”.

¶ PSFS: scores were from 0 to 10, with 0 indicating “unable to perform” and 10 indicating “able to perform at prior level”. PSFS data were not available for one participant in the placebo group.

** SF-12: scores were from 0 to 100, with higher scores indicating better quality of life.

ATRS, Achilles Tendon Rupture Score; PRP, platelet-rich plasma; PSFS, Patient-Specific Functional Scale; SD, standard deviation; SF-12, 12-Item Short-Form Health Survey questionnaire.

Two-year questionnaires were sent to the 216 participants (109 placebo, 107 PRP) who had completed a 24-week questionnaire and had not died or withdrawn consent. Of these, 182 returned their questionnaires (94 placebo, 88 PRP), a response rate of 84%. Overall, 182/230 (79%) randomized participants provided two-year responses. Of the responding participants, 7.1% (13/182) were contacted by telephone rather than by questionnaire.

An attending surgeon delivered the injections for 86/113 (76%) of the PRP group and 87/116 (75%) of the placebo group. Surgical residents/fellows or specialist physiotherapists delivered the remaining injections. Injections were delivered a mean 5.3 days (SD 3.0) post-injury. Further detail on PRP analysis is available elsewhere;^17^ in summary, the PRP prepared had 4.1-fold (95% confidence interval (CI) 3.6 to 4.5) greater platelet concentrations and 2.2-fold (95% CI 2.0 to 2.5) greater leucocyte concentrations than whole blood. Platelet quality measurements showed that PRP was not activated during preparation.

**Fig. 2:**
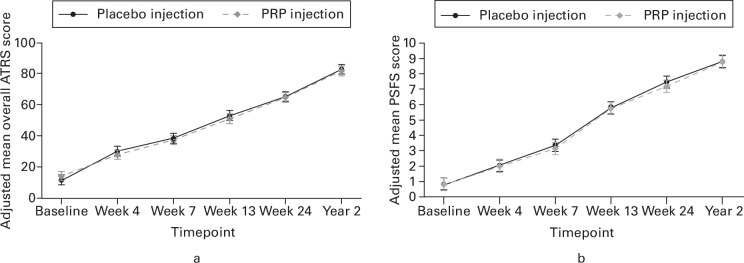
Results from the adjusted repeated measures mixed effects regression model demonstrating the change in: a) Achilles Tendon Rupture Score (ATRS) (scores from 0 to 100, with 0 indicating “major limitations” and 100 indicating “no limitations”); and b) Patient-Specific Functional Scale (PSFS) (scores from 0 to 10, with 0 indicating “unable to perform” and 10 indicating “able to perform at prior level”) in platelet-rich plasma (PRP) injection and placebo injection patients over time. The error bars represent 95% confidence intervals.

**Fig. 3:**
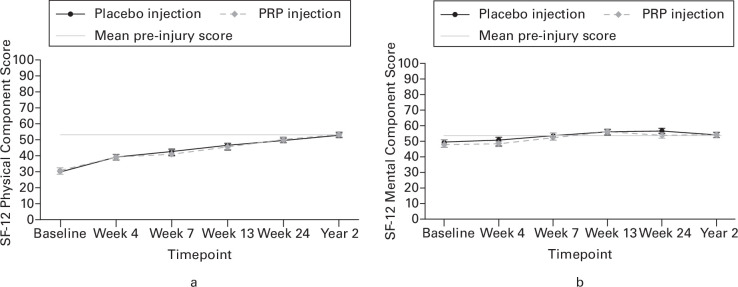
Results from the adjusted repeated measures mixed effects regression model demonstrating the change in 12-Item Short-Form Health Survey questionnaire (SF-12): a) Physical; and b) Mental Component Score in platelet-rich plasma (PRP) injection and placebo injection patients over time, accounting for pre-injury scores.

Mean ATRS scores at two years were 82.2 (SD 18.3) in the PRP group (n = 85) and 83.8 (SD 16.0) in the placebo group (n = 92). There was no evidence of differences between the PRP and placebo groups in the patient-reported outcomes of the ATRS, ATRS pain score, PSFS, or SF-12 at two years (Table II). Overall, ATRS (Figure 2a), PSFS (Figure 2b), and SF-12 (Figure 3) scores increased from baseline to two-year post-randomization at consistent rates in both the PRP injection group and the placebo injection group, irrespective of stratification and other prognostic variables taken into account.

**Table II.** Patient-reported outcomes at two-year follow-up.

Outcome	n*	PRP injection, mean overall ATRS (95% CI)	Placebo injection, mean overall ATRS (95% CI)	Mean difference (SE; 95% CI)	p-value
ATRS at 2 yrs adjusted† ‡	177	82.371 (78.909 to 85.832)	83.123 (79.841 to 86.405)	-0.752 (2.434; -5.523 to 4.020)	0.757
ATRS CACE: Treatment compliance†	177			-1.954 (2.783; -7.408 to 3.500)	0.482
ATRS CACE: PRP quality compliance†	177			-2.033 (2.900; -7.715 to 3.650)	0.483
ATRS pain scale at 2 yrs¶ §	181	Median 10 (IQR 8 to 10)	Median 9 (IQR 9 to 10)		0.848
PSFS at 2 yrs adjusted‡ **	177	8.777 (8.346 to 9.209)	8.800 (8.389 to 9.211)	-0.023 (0.304; -0.618 to 0.573)	0.941
SF-12 Physical Component at 2 yrs adjusted‡ ††	177	53.184 (51.603 to 54.764)	52.778 (51.276 to 54.281)	0.405 (1.114; -1.778 to 2.588)	0.716
SF-12 Mental Component at 2 yrs adjusted‡	177	53.964 (52.070 to 55.859)	54.119 (52.321 to 55.917)	-0.154 (1.333; -2.767 to 2.459)	0.908

* Number of individuals included in the model, who provided a two-year outcome.

† ATRS scores from 0 to 100, with 0 indicating “major limitations” and 100 indicating “no limitations”.

‡ Repeated measures mixed effects regression model with: outcome as the dependent variable; treatment group, study site, and age category as fixed effects; and time elapsed included as a random effect.

§ ATRS pain scale from 0 to 10, with 0 indicating “major limitations” and 10 indicating “no limitations”.

¶ ATRS pain was analyzed using Spearman’s rank correlation, due to most participants having reached a score of ten points at two years.

** PSFS scores from 0 to 10, with 0 indicating “unable to perform” and 10 indicating “able to perform at prior level”.

†† SF-12 scores were from 0 to 100, with 0 indicating worst and 100 indicating best.

ATRS, Achilles Tendon Rupture Score; CACE, compliance average causal effects; CI, confidence interval; IQR, interquartile range; PRP, platelet-rich plasma; PSFS, Patient-Specific Functional Scale; SE, standard error; SF-12, 12-Item Short-Form Health Survey questionnaire.

**Fig. 4:**
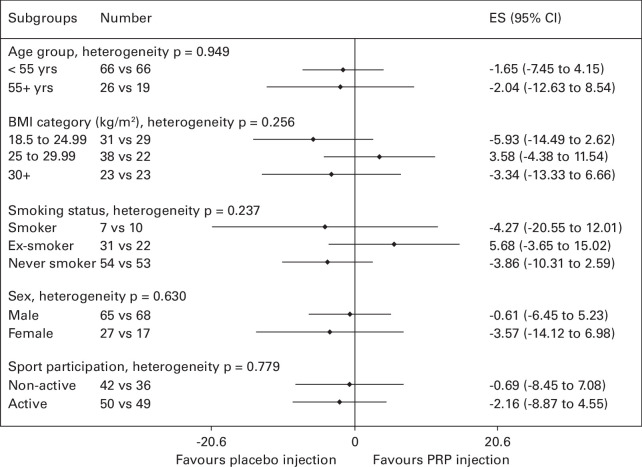
Forest plot demonstrating the effect (mean difference with 95% confidence interval (CI)) of intervention on overall Achilles Tendon Rupture Score in subgroups of defined stratification and prognostic factors. *Number in placebo injection arm versus number in platelet-rich plasma (PRP) injection arm. ES, effect size.

Consistency of treatment effect for ATRS was explored for subgroups of age, BMI, smoking, sex, and sports participation (Figure 4). There was low variability between subgroups, with all remaining close to a zero difference between PRP injection and placebo injection for overall ATRS score.

A total of 78 participants who received the allocated PRP injection and were not lost to follow-up at two years also completed their final ATRS assessment. Seven participants who received a placebo injection, in place of the PRP they were allocated to, completed their two-year follow-up ATRS. This resulted in a 92% (78/85) compliance to treatment allocation among PRP patients within the trial. In the subsequent CACE analyses for treatment compliance and for treatment compliance with high-quality PRP, there was no evidence of a difference between the PRP and placebo groups, consistent with the ITT analysis (Table II).

Neither PRP cellular or growth factor (IGF-1, TGF-β1, PDGF-AB, VEGF, and FGFb) concentrations or quality measurements (activated CD62p and mean fluorescence intensity (MFI)) correlated with the two-year ATRS (Table III). Of the 182 participants who completed the long-term follow-up, none reported a re-rupture of their Achilles tendon between 24 weeks and two years.

**Table III.** Platelet-rich plasma quality, cellular and growth factor correlations with Achilles Tendon Rupture Score at two years.

Parameter	n	r	% variance*	p-value†
**Cell count**				
Red blood cell count	77	0.011	0.011	0.927
White blood cell count	77	< 0.001	< 0.001	0.996
Platelet count‡	74	0.065	0.419	0.584
**Platelet quality**				
Activated CD62p expression (%)	77	-0.119	1.411	0.303
Mean fluorescence intensity	77	0.025	0.065	0.826
**Growth factor**				
IGF-1	78	0.059	0.347	0.608
TGF-β1	75	0.0672	0.452	0.567
PDGF-AB	76	-0.076	0.580	0.513
VEGF	78	-0.165	2.706	0.150
FGFb	78	0.041	1.168	0.722

* Proportion of variance in Achilles Tendon Rupture Score at two years explained by key blood parameter, calculated as: ((r × r) × 100).

† Pearson’s correlation.

‡ As PLT-F (the Fluorescent Platelet Count on the Sysmex XN analyser).

FGFb, basic fibroblast growth factor; IGF-1, insulin-like growth factor-1; PDGF-AB, platelet-derived growth factor AB; TGF-β, transforming growth factor-beta; VEGF, vascular endothelial growth factor.

## Discussion

The PATH-2 trial two-year follow-up found no evidence that PRP improved Achilles tendon symptoms and function, pain, goal attainment, and quality of life in patients with Achilles tendon ruptures. These findings question the hypothesis that PRP affects the quality of the recovering tendon, in terms of resulting in improvements in longer-term recovery outcomes. This finding is supported by the good follow-up rate, robust trial methods and conduct, comparability between the characteristics of responders and non-responders, and consistency across outcome domains.

Previous under-powered trials of PRP in Achilles rupture were hampered by a lack of standardization in the PRP preparation methods and quality control procedures, small sample sizes, potential confounding effects of use with surgery, and short-term follow-up.^24-26^ Along with the 24-week follow-up reported previously,^10^ this study provides robust evidence to question the clinical efficacy and value of PRP for acute Achilles ruptures in both shorter-term and longer-term recovery.

The trajectory of recovery over two years showed improvements in patient-reported outcomes over time. There was evidence from pre-injury physical and mental quality of life (SF-12) scores, completed by recall during the acute trauma clinic visit within 12 days of injury, that participants were close to being fully recovered. However, this contrasts with the Achilles-specific symptoms and function assessed by the ATRS, which indicates deficits at two years. There were no pre-injury ATRS scores, which limits interpretation, but it could be anticipated that further recovery is possible after two years given the recovery at seven years reported by Brorsson et al.^27^

One important feature of the PATH-2 trial was the quality control measurements of all the PRP preparations that were injected into all participants,^10,17^ in line with modern guidelines.^28,29^ These quality control analyses revealed that the treated patients received a single injection of good-quality leucocyte-rich PRP (L-PRP), all prepared by the same methodology and device (MAG 200 MAGELLAN) across all trial centres. The platelet, leucocyte, and erythrocyte concentrations observed within the L-PRP preparations were not only in the same order of magnitude as previously reported studies, but they were in line with manufacturer specifications using the Magellan device.^30,31^ As expected, there were also significant associations between the concentrations of growth factors within the L-PRP with both platelet and leucocyte counts.^17^ Furthermore, the growth factor concentrations obtained were also comparable and within the same order of magnitude of previously reported levels using the same device.^32,33^ Remarkably, variations in L-PRP content (cell counts, growth factor content, or platelet quality) were not associated with the level of recovery of Achilles tendon-muscle function at 24 weeks or with patient-reported recovery at two years.^10^ Interestingly, a small number of participants also received very low concentrations of platelets and growth factors within their PRP due to a few unforeseen problems with the Magellan device, but this had no notable impact on recovery. A few patients also received very high concentrations of platelets, but this also had no positive or negative impact upon outcomes at 24 weeks or two years. Despite this, the biological effects of the leucocytes and red cells in L-PRP for tendon healing remain uncertain, with in vitro investigations potentially identifying both a range of positive or negative effects.^34-36^ Currently, leucocyte-rich preparations are widely used in clinical practice as buffy coat derived PRP results in leucocyte and red cell inclusion within L-PRP preparations. Future studies comparing different formulations including L-PRP and PRP both with and without red blood cells are therefore required.^37^

The PATH-2 trial limitations included that the volumes of whole blood taken from the two randomization groups were different (55 ml PRP vs 5 ml placebo). However, we would have anticipated any resentful demoralization from the placebo group participants upon realizing that they were not having the experimental intervention, to have only increased the likelihood of showing a difference in favour of PRP. We did not use the HRET for the two-year follow-up, the primary endpoint for the 24-week follow-up, as it required a hospital visit and specialist equipment and training over the 19 hospitals, which was beyond the resources available for this longer-term follow-up study. Deficits in performance in the HRET can be present at two years or more after Achilles rupture, so this measure could have provided greater sensitivity as an outcome measure.^27^

In conclusion, PRP injections did not improve patient-reported function or quality of life two years after acute Achilles tendon rupture compared with placebo, indicating that PRP offers no patient benefit in the long term.

**Take home message**

- Autologous platelet-rich plasma (PRP) containing supraphysiological platelet concentrations from whole blood are used to treat acute Achilles ruptures with the aim of improving tendon healing, which may result in improvement in longer-term outcomes.

- The PATH-2 trial found no evidence of efficacy at 24 weeks after treatment, but there is uncertainty about clinical efficacy in the longer term.

- Our study found no evidence that PRP injection improved patient-reported function or quality of life two years after acute Achilles tendon rupture compared to placebo, and therefore offers no patient benefit in the longer term.
